# Inhibition of Notch activity promotes pancreatic cytokeratin 5-positive cell differentiation to beta cells and improves glucose homeostasis following acute pancreatitis

**DOI:** 10.1038/s41419-021-04160-2

**Published:** 2021-09-23

**Authors:** Xiaoyi Zhang, Jing Tao, Jia Yu, Ning Hu, Xuanzhe Zhang, Guirong Wang, Jiarui Feng, Xingcheng Xiong, Man Li, Dongqi Chai, Hanjun Li, Yuping Rong, Zhigang Tang, Weixing Wang, Zhiyong Peng, Qiao Shi

**Affiliations:** 1 Department of Critical Care Medicine, Zhongnan Hospital of Wuhan University, Wuhan, China; 2 Department of General Surgery, Renmin Hospital of Wuhan University, Wuhan, China; 3 Department of Pancreatic Surgery, Renmin Hospital of Wuhan University, Wuhan, China; 4 Central Laboratory, Renmin Hospital of Wuhan University, Wuhan, China; 5 Department of Surgery, SUNY Upstate Medical University, Syracuse, USA; 6 Department of Medical Management, Renmin Hospital of Wuhan University, Wuhan, China; 7 Hubei Key Laboratory of Digestive System Disease, Renmin Hospital of Wuhan University, Wuhan, China

**Keywords:** Endocrine system and metabolic diseases, Pancreatitis

## Abstract

Some individuals develop prediabetes and/or diabetes following acute pancreatitis (AP). AP-induced beta-cell injury and the limited regenerative capacity of beta cells might account for pancreatic endocrine insufficiency. Previously, we found that only a few pancreatic cytokeratin 5 positive (Krt5^+^) cells differentiated into beta cells in the murine AP model, which was insufficient to maintain glucose homeostasis. Notch signaling determines pancreatic progenitor differentiation in pancreas development. This study aimed to examine whether Notch signaling inhibition could promote pancreatic Krt5^+^ cell differentiation into beta cells and improve glucose homeostasis following AP. Pancreatic tissues from patients with acute necrotizing pancreatitis (ANP) were used to evaluate beta-cell injury, Krt5^+^ cell activation and differentiation, and Notch activity. The murine AP model was induced by cerulein, and the effect of Notch inhibition on Krt5^+^ cell differentiation was evaluated both in vivo and in vitro. The results demonstrated beta-cell loss in ANP patients and AP mice. Krt5^+^ cells were activated in ANP pancreases along with persistently elevated Notch activity, which resulted in the formation of massive duct-like structures. AP mice that received Notch inhibitor showed that impaired glucose tolerance was reversed 7 and 15 days following AP, and increased numbers of newborn small islets due to increased differentiation of Krt5^+^ cells to beta cells to some extent. In addition, Krt5^+^ cells isolated from AP mice showed increased differentiation to beta cells by Notch inhibition. Collectively, these findings suggest that beta-cell loss contributes to pancreatic endocrine insufficiency following AP, and inhibition of Notch activity promotes pancreatic Krt5^+^ cell differentiation to beta cells and improves glucose homeostasis. The findings from this study may shed light on the potential treatment of prediabetes/diabetes following AP.

## Introduction

Diabetes mellitus (DM) affectes over 300 million people worldwide and poses an enormous burden on society [1]. Diabetes secondary to exocrine pancreatic diseases, including acute pancreatitis (AP), chronic pancreatitis and pancreatic tumor, is referred to as type 3c diabetes [2]. Recent studies demonstrated that 37%-59% of individuals develop prediabetes and/or DM after AP [3, 4], which might be attributed to beta-cell injury [5]. Unlike the exocrine pancreas, which possesses an extensive regenerative capacity, studies have indicated the limited capacity of beta-cell regeneration in the natural course of pancreatic diseases [6, 7]. Thus promoting beta-cell regeneration and the recovery of endocrine function following AP is of great importance.

Among the strategies to promote beta-cell regeneration, facilitating beta-cell proliferation, converting other endocrine lineages to beta cells and seeking pancreatic stem cells or progenitors to stimulate the regeneration of beta cells in vivo appear to be reliable and promising [1, 8, 9]. Krt5^+^ cells have been demonstrated to play a role as stem cells and to facilitate regeneration in the lung and submandibular gland [10–12]. In our previous study, we observed that Krt5^+^ cells were activated in the injured pancreas of the murine AP model, and only a few could differentiate toward beta cells. The limited differentiation cannot maintain glucose homeostasis after AP [13]. Thus, strategies promoting Krt5^+^ cell differentiation to functional beta cells would be promising to avoid/delay the onset of DM following AP.

Canonical Notch signaling is a relatively conservative pathway that regulates cell fate determination during development and maintains adult tissue homeostasis through interactions of receptors (Notch1–4) and ligands (Jagged1/2, Dll1/3/4) [14, 15]. When Notch pathway is activated, the intracellular domain (NICD) of the receptors released from the cell membrane, which translocates to the nucleus and binds to the DNA binding receptor Rbpj, resulting in subsequent activation of the Notch target genes, such as Hes family members [16]. Rbpj is a key mediator of Notch signaling, and it associates with all four types of Notch receptors [17]. The transcriptional factor Hes1 is a main target of the Notch signaling pathway, which expresses in progenitor cells in the embryonic stage, and is thought to maintain the progenitor state of these cells [18]. Previous studies in pancreatic development have mapped out that Notch signaling determines the endocrine or exocrine fates of pancreatic progenitors [19]. Mice deficient in the Notch intracellular transcription effector Rbpj or downstream target Hes1 showed accelerated differentiation of pancreatic endocrine cells [20, 21]. Therefore, promoting the differentiation of more pancreatic Krt5^+^ cells into functional beta cells through the regulation of Notch activity might be a novel strategy to reduce the incidence of prediabetes/diabetes after AP.

## Materials and methods

### Human subjects

The use of human pancreases in our study was approved by the Institutional Review Board of Renmin Hospital of Wuhan University (WDRY2018-K063), and was in accordance with the principles of the Declaration of Helsinki II. Acute necrotizing pancreatitis (ANP) specimens were collected during surgery for necrotic tissue removal procedures in ANP patients. Relatively normal (Control, CON) pancreatic specimens were harvested from patients who were diagnosed with traumatic pancreatic rupture, duodenal papillary adenocarcinoma (DPA), or solid pseudopapillary neoplasm (SPN) of the pancreas that required surgery. These control pancreatic specimens were collected away from the tumor or traumatized tissue. Pancreas from a brain-dead donor for organ transplantation was also obtained as control. All included patients were ensured to have no previous DM prior to hospitalization. The pancreases from six patients with ANP (A1-A6) and six controls (C1-C6) were used. Detailed clinical information is listed in Supplementary Table 1. The obtained pancreatic tissues were embedded in paraffin or frozen at −80 °C for further histological and biochemical analyses.

### Animal experiments

The animal experiments were approved by the Institutional Review Board of Renmin Hospital of Wuhan University (WDRM 20190108). FVB/N mice were purchased from Beijing Vital River Laboratory Animal Technology Co., Ltd. (Beijing, China) and were housed in pathogen-free conditions under a 12-h light/dark cycle with free access to standard rodent chow and water. The AP model was established by six hourly intraperitoneal injections of cerulein (100 μg/kg body weight, Sigma-Aldrich, USA), dissolved in 0.9% saline administered on four consecutive days [13]. The control group received intraperitoneal injections of sterile saline instead. For Notch signaling inhibition, DAPT (GSI-IX), which is a γ-secretase inhibitor, was dissolved in corn oil with 4% dimethylsulfoxide (DMSO). DAPT (50 mg/kg body weight, Selleck, USA) was administered on the same day following the last cerulein injection and continued for another six consecutive days (the AP1d + DAPT group received one dose of DAPT, the AP3d + DAPT group received three doses of DAPT, and the AP7d + DAPT and AP15d + DAPT groups received seven doses of DAPT). Mice were sacrificed on days 1, 3, 7, and 15 following AP model establishment. Pancreatic tissues were embedded in paraffin or frozen at −80 °C for further analyses.

### Fasting glucose (FG) and intraperitoneal glucose tolerance test (IPGTT)

After overnight fasting for 16 h, FG levels were measured with glucometer (Onetouch UltraVue, Johnson & Johnson, USA) through the tail vein. IPGTT was performed by intraperitoneal injection of 2 g/kg glucose. Then blood glucose levels at 15, 30, 60, 90, and 120 min following glucose injection were measured via the tail vein.

### Histologic study

Paraffin-embedded human and mouse pancreases were sliced into 4 μm sections and subjected to hematoxylin and eosin (H&E) staining. Pathologic parameters including edema, acinar necrosis, hemorrhage and fat necrosis, inflammation and perivascular infiltration were carefully scored by two researchers independently according to scoring criteria [22].

### Primary cell culture

FVB/N mice following AP establishment or control mice were killed by cervical dislocation and immersed in 75% ethanol for sterilization. A midline incision was made in the abdomen, and the abdominal cavity was exposed. Then, pancreatic tissue was harvested and transferred to a sterile Petri dish, and the pancreas was sliced into small pieces. Then, pancreas slices were transferred into a 50 ml polypropylene tube containing collagenase XI dissolved in 1×HBSS (1.25 mg/ml) and incubated in a 37 °C water bath for 15 min with gentle shaking. During this period, mechanical dissociation was performed with 5 ml pipettes by moving the pancreas fragments back-and-forth. After the pancreatic tissue was well digested, CaCl_2_ solution (1 mM) was added to stop the enzymatic reaction, and the tube was centrifuged at 290×*g* and 4 °C for 2 min. Then, the supernatant was carefully aspirated and discarded. The pellet was resuspended in cold CaCl_2_ solution followed by centrifugation at 290×*g* and 4 °C for 2 min. Then the supernatant was discarded, and this step was repeated twice. The suspension was then filtered into a new 50 ml tube with a 70 μm filter to remove islets. The filtration was then centrifuged and resuspended in RPMI 1640 medium supplemented with 12% serum replacement for ESCs/iPSCs (KnockOut SR, 10828-028, Gibco, USA), 100 IU/ml penicillin, 100 μg/ml streptomycin and 20 mM L-glutamine. The isolated cells were then seeded into culture dishes, small islets were carefully removed under a microscope, and the cells were cultured at 37 °C under a 5% CO_2_ atmosphere. The medium was changed within 24 h to remove the remaining pancreatic acini and islets. For Notch inhibition, a range of concentrations of DAPT were supplemented.

### Immunofluorescence

For immunofluorescent staining of human and mouse pancreases, pancreatic sections were deparaffinized and rehydrated. Tris-EDTA (pH 9.0, Servicebio, China) buffer was used for antigen retrieval at the boiling point for 10 min in a microwave. For primary cells growing on the glass slides, the slides were washed with PBS twice and then fixed in 4% paraformaldehyde for 15 min at room temperature. Following permeabilization with 0.02% Triton X-100 (Sigma-Aldrich, St. Louis, USA) in PBS for 45 min at room temperature, the tissue sections and cell slides were blocked in 10% donkey serum for 1 h at room temperature. Then slides were incubated with primary antibodies diluted in PBS overnight at 4 °C. Alexa-conjugated secondary antibodies were used for fluorescent detection. Finally, slides were mounted in mounting medium with DAPI (ab104139, Abcam, USA). The double-labeling immunofluorescence staining method was used to prove the differentiation of Krt5^+^ cells into beta cells. This method has been used to prove alpha cells differentiation into beta cells and islet pericytes converting into myofibroblasts in the pancreas [23, 24]. Insulin-stained sections were used for small islet determination. Single beta cells and clusters up to five beta cells were counted as small newborn islets, as previously reported [25]. The following antibodies were used: anti-pancreatic alpha amylase (1:200, Ab21556, Abcam), anti-insulin (1:800, Sc-7838, Santa Cruz Biotechnology), anti-insulin (1:400, 3014s, Cell Signaling Technology), anti-cytokeratin 5 (1:100, Ab53121, Abcam), anti-Hes1 (1:200, Ab71559, Abcam), anti-Glut2 (1:200, abs119876, Absin), anti-Glucagon (1:200, 15954-1-AP, Proteintech), anti-Somatostatin (1:200, 17512-1-AP, Proteintech), anti-Pancreatic polypeptide (1:1000, ab272732, Abcam), Donkey anti-rabbit IgG H&L (Alexa Fluor^®^ 488) (1:200, Ab150073, Abcam), and Donkey anti-goat IgG (H + L) Alexa Fluor 594 (1:200, A-11058, Thermo Fisher Scientific).

### TUNEL and immunofluorescence colabeling

Colabeling of TUNEL and insulin was performed by using a Click-iT Plus TUNEL Assay kit (C10618, Invitrogen, USA) according to the manufacturer’s instructions with a slight modification. Pancreatic sections were deparaffinized and rehydrated. Then slides were fixed in 4% paraformaldehyde for 15 min, followed by permeabilization with proteinase K. Slides were rinsed in PBS for 5 min and immersed slides in 4% paraformaldehyde for 5 min at 37 °C. Then, TdT buffer was added to pancreatic sections and incubated at 37 °C for 10 min. Then, slides were incubated with TdT reaction mixture and rabbit anti-insulin antibody (1:400, 3014s, Cell Signaling Technology) at 4 °C overnight. Then, slides were rinsed with 3% BSA and 0.1% Triton X-100 in PBS. Click-It Plus TUNEL reaction cocktail and Donkey anti-rabbit IgG H&L (Alexa Fluor® 488) (1:200, Ab150073, Abcam) were added to sections and incubated for 30 min at 37 °C protected from light. Then, slides were mounted in DAPI with mounting medium.

### Immunohistochemistry

An immunohistochemistry kit (PV-9001, ZSGB-Bio, China) was used for immunohistochemistry assays. Deparaffinization, rehydration and antigen unmasking of pancreatic slides were the same as those of the immunofluorescent staining procedures. Citrate buffer (pH 6.0, Servicebio, China) was used for antigen recovery. Slides were blocked with endogenous peroxidase blocker for 10 min at 37 °C and then incubated at 4 °C overnight with primary antibodies. Response enhancer was added to pancreatic sections and incubated for 20 min at 37 °C. Then, slides were incubated with goat anti-rabbit HRP-conjugated antibody for hybridization at 37 °C for 30 min. DAB (ZLI-9017, ZSGB-Bio, China) or AEC (ZLI-9036, ZSGB-Bio, China) substrate was used to develop color in pancreatic sections. Then, the slides were counterstained with hematoxylin (G1004-500, Servicebio, China) and dehydrated sections. Finally, slides were mounted with neutral balsam mounting medium or glycerol jelly mounting medium. The following antibodies were used: anti-cleaved caspase 3 (1:400, 9661s, Cell Signaling Technology), anti-cytokeratin 5 (1:100, Ab53121, Abcam), anti-Rbpj (1:200, 5313s, Cell Signaling Technology), and anti-Hes1 (1:200, Ab71559, Abcam).

### Western blotting

Pancreatic tissues were homogenized with a freezing grinder in radioimmunoassay precipitation (RIPA) buffer (P0013B, Beyotime, China) supplemented with proteinase and phosphatase inhibitors (04693132001 and 0490684500, Roche, Switzerland). After centrifugation (12,000 rpm, 10 min, 4 °C) to remove debris, supernatants were collected and used for immunoblotting analysis. For primary cells, cells were scraped off the culture dish and incubated with RIPA lysis buffer, sonicated, and spun at 12 000 rpm and 4 °C for 10 min to remove debris. The total protein concentration was determined using a BCA protein assay kit (23227, Thermo Scientific, USA). Equal amounts of protein (20 μg) were separated by SDS-PAGE and transferred onto PVDF membranes. Membranes were blocked with 10% silk milk (G5002, Servicebio, China) diluted in TBST at room temperature for 1 h and probed with primary antibodies. Rabbit anti-GAPDH antibody was used as a control for protein loading. Antigen-antibody complexes were probed with HRP-conjugated secondary antibody for 1 h at room temperature. Immunoproducts were detected using an Ultra-sensitive ECL Chemiluminescence Kit (P0018FS, Beyotime, China). Protein expression levels were quantified using Quantity One software (v4.6.2, Bio-Rad Laboratories, Inc., USA). The following antibodies were used: anti-cytokeratin 5 (1:1000, Ab53121, Abcam), anti-Rbpj (1:1000, 5313s, Cell Signaling Technology), anti-Hes1 (1:1000, 11988s, Cell Signaling Technology), anti-cleaved caspase-3 (1:1000, 9661T, Cell Signaling Technology), anti-GAPDH (1:2000, Abs132004, Absin), and HRP-conjugated goat anti-rabbit IgG (1:5000, GB23303, Servicebio).

### Measurement of insulin content

Cells treated with DAPT for 72 h were scraped off the culture dish and incubated with RIPA lysis buffer, sonicated, and spun at 12,000 rpm and 4 °C for 10 min to remove debris. The supernatants were collected to carry out ELISA. The commercial kit (E-EL-M2614c, Elabscience, China) used in our study used the sandwich ELISA principle. The plate was precoated with an antibody specific to mouse insulin. The standard working solution and diluted samples (100 μL) were added to the plate and incubated for 90 min at 37 °C. Then, the liquid was removed from each well, and 100 μL biotinylated detection antibody working solution was added and incubated for 1 h at 37 °C. After washing the plate three times with washing buffer, 100 μL HRP conjugate working solution was added to each well and incubated for 30 min at 37 °C. The wash process was repeated five times, and 90 μL of substrate reagent was then added to each well and incubated for 15 min at 37 °C protected from light. Finally, 50 μL of stop solution was added to each well to stop the reaction, and the optical density of each well was determined using a microplate reader set to 450 nm. A standard curve was established, and the insulin concentrations of the samples were calculated.

### Statistical analysis

Data were presented as mean ± SEM and were analyzed by SPSS 19.0 (IBM, USA). An independent two-tailed t-test was used to compare differences between the two groups, while two-way ANOVA analysis was applied to comparison among several groups. Pearson regression analyses were used to analyze the correlation between the Krt5^+^ area and injury scores. A *P* value < 0.05 was considered statistically significant.

## Results

### Beta-cell loss in pancreases of ANP patients

**Fig. 1:**
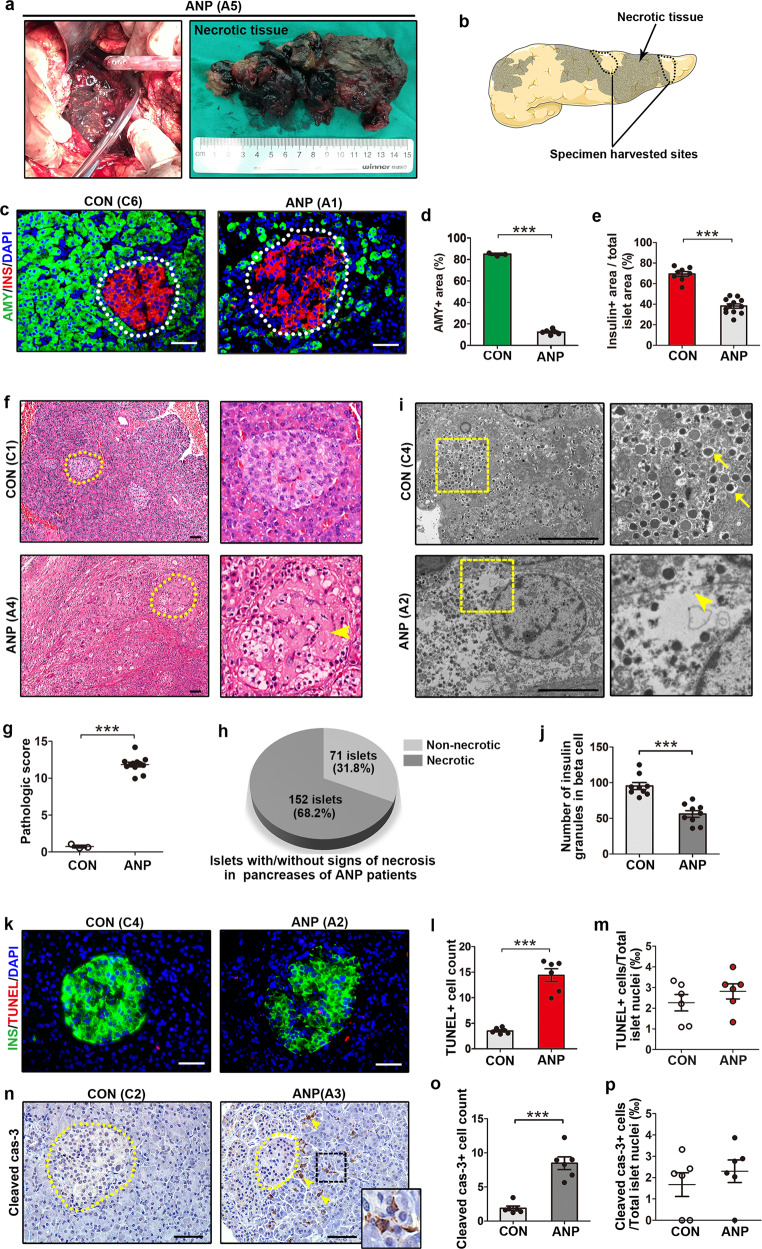
Beta-cell loss in pancreases of ANP patients. **a** Surgical removal of pancreatic and peripancreatic necrotic tissues in ANP patients. **b** Schematic depicting that specimens of ANP patients in our study were fresh pancreatic tissues in adjacent to surgical margin. **c** Immunofluorescent graphs of pancreases from control and ANP individuals immunostained for amylase (green), insulin (red) and DAPI (blue). **d** Quantification of pancreatic area immunostained positive for amylase (shown as %). **e** Quantification of islet area labeled with insulin divided by total islet area (shown as %) in pancreatic sections from control individuals and ANP patients. To perform insulin area calculation, pancreatic tissues were examined across the sections, 20.55 ± 1.82 islets were analyzed per section. **f** Representative H&E staining of pancreases from control individuals and ANP patients, and magnification of islets. Arrowhead points to the necrotic part. **g** Quantification of pancreatic tissue injury in pancreases from control and ANP individuals (*n* = 3 in the control group, *n* = 6 in ANP group with 2 sections per patient). **h** A pie chart depicting that of 223 islets in six ANP patients (2 sections per patient), 152 islets showed signs of cell necrosis on H&E staining. **i** Representative electron microscopy graphs showing beta cells of control and ANP individuals. Arrows point to insulin granules; arrowhead points to dismantled cell membranes. **j** Quantification of insulin granules in beta cells of ANP and control individuals under ×5000 magnification (*n* = 3 in each group with 3 beta cells per individual). **k** Representative images of pancreases from control individuals and ANP patients, stained for TUNEL (red), insulin (green) and DAPI (blue). **l** Quantification of TUNEL^+^ cells in pancreases of control and ANP individuals (×200 magnification). **m** Quantification of TUNEL^+^ beta cells divided by total islet cells (shown as ‰) in islets from control and ANP individuals. To perform TUNEL^+^ cell counting in islets, ten islets were randomly picked across each section. **n** Representative staining of cleaved caspase-3 in pancreases of control and ANP individuals. Arrowheads point to cleaved caspase-3^+^ cells. **o** Quantification of cleaved caspase-3^+^ cells in control and ANP sections under ×200 magnification. **p** Quantification of cleaved caspase-3^+^ cells in islets divided by total islet cells (shown as ‰) from control and ANP individuals. To perform cleaved caspase-3^+^ cell counting in islets, ten islets were randomly determined across each section. AMY, amylase; ANP, acute necrotizing pancreatitis; CON, control; INS, insulin. Data are mean ± SEM, *n* = 3–6. ***p < 0.001. Scale bars, 50 μm (**c**, **f**, **k**, **n**) and 5 μm (**i**).

In clinical settings, there is massive pancreatic tissue necrosis in some patients with ANP, which requires the removal of necrotic tissues to control infection (Fig. 1a). The ANP specimens used in our study were fresh tissues harvested adjacent to the surgical margin (Fig. 1b). Immnostaining of amylase demonstrated significantly reduced acinar area in ANP pancreases (Fig. 1c, d), which was consistent with extensive pancreas destruction on H&E staining (Fig. 1f). The insulin-positive area, which represents beta-cell mass, was also decreased in ANP pancreases (Fig. 1c, e). To analyze the causes of beta-cell loss, H&E staining of sections was carefully examined. There was little tissue damage or inflammation in any case of the control group. Remarkable edema and acinar necrosis, hemorrhage and fat necrosis accompanied by significant inflammatory cell infiltration were observed in pancreatic sections of ANP patients (Fig. 1f, g). Compared to the integral and regular structures of islets in the controls, irregularly structured islets and cell necrosis features, including disappearance of the nucleus, vacuolization and dismantling of the cytoplasm, were observed in ANP pancreases. We then analyzed 223 islets in six ANP patients (2 sections per patient) by H&E staining, among which 152 islets showed the features of cell necrosis in islets (Fig. 1h). In addition, electron microscopy analysis revealed cell death manifestations, including dismantled cell membranes and cytoplasm, autophagic vacuoles, and decreased numbers of insulin-secreting granules in beta cells of ANP pancreases (Fig. 1i, j and Supplementary Fig. 1). Then, a TUNEL assay was performed to evaluate apoptosis in the pancreases of both ANP and control individuals. Although significantly increased TUNEL positive cells were observed in pancreatic sections of ANP patients (Fig. 1k, l), we did not notice increased apoptosis of beta cells (Fig. 1m). Immunostaining of cleaved caspase-3 also showed increased apoptosis in the exocrine pancreas but not in the islets of ANP patients (Fig. 1n–p). In addition, immunostaining of other endocrine cells did not show significantly decreased proportions in the ANP specimens (Supplementary Fig. 2).

### Extensive Krt5^+^ cell activation in ANP correlates with pancreatic injury

**Fig. 2:**
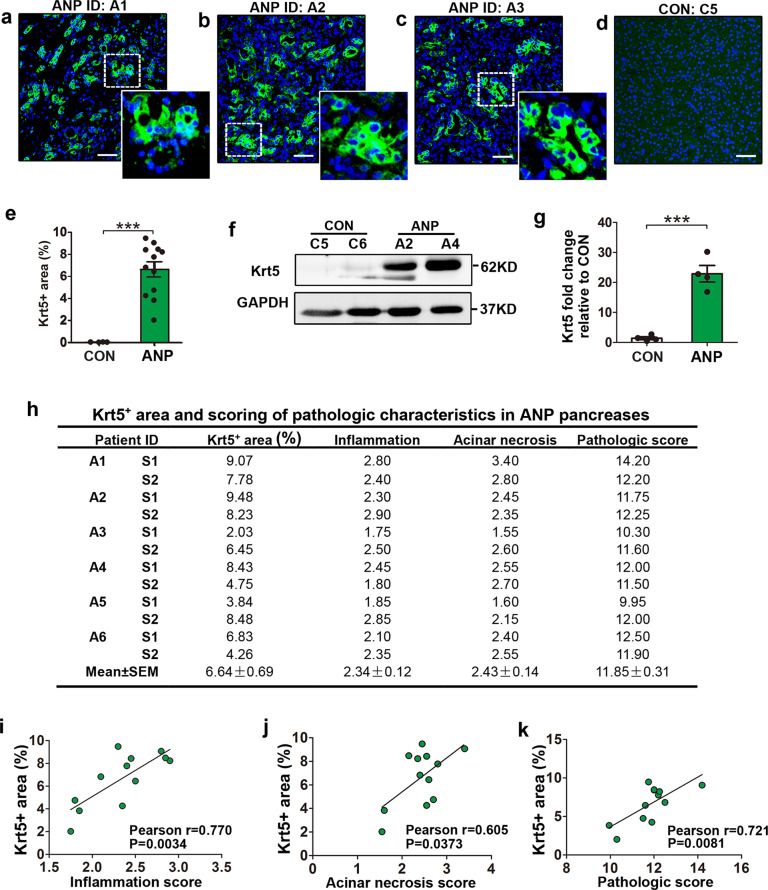
Krt5^+^ cells are activated in pancreases of ANP patients and correlate with the pancreatic injury. **a**–**d** Representative images of pancreases from ANP and control individuals immunostained with Krt5 (green) and DAPI (blue) showing massive Krt5^+^ cells activation following ANP. **e** Quantification of the area covered with Krt5^+^ cells in control and ANP pancreases, divided by the total area (shown as %, *n* = 4 in control group, *n* = 6 in ANP group with 2 sections per ANP patient). **f** Representative western blots of Krt5 expression in pancreases from control and ANP individuals. **g** Quantification of western blots for Krt5 fold change in ANP pancreases compared with that in control pancreases. **h** Krt5^+^ area (%) and scoring of pathologic characteristics of pancreases from ANP patients. Two sections per patient were analyzed. **i**–**k** Pearson regression analyses showing the correlation between the Krt5^+^ area and inflammation score (**i**), acinar necrosis score (**j**) and pathologic score (**k**). ANP, acute necrotic pancreatitis; CON, control. Data are mean ± SEM, *n* = 4–6. ***p < 0.001. Scale bars, 50 μm.

Our previous study has demonstrated Krt5^+^ cell activation in a murine AP model [13]. To assess Krt5^+^ cell activation in the human pancreas, we performed immunofluorescence staining for Krt5 in the pancreases of both ANP and control individuals. Remarkably increased Krt5^+^ cells were observed in ANP pancreases compared with control individuals, in which rare Krt5^+^ cells were noted (Fig. 2a–e). These Krt5^+^ cells clustered in small colonies or formed duct-like structures, and scattered single Krt5^+^ cells could also be observed. Western blotting demonstrated significantly increased Krt5 levels in pancreatic tissues of ANP patients compared to controls (Fig. 2f, g). We further assessed whether pathological parameters might be correlated with the expansion of Krt5^+^ cells (Fig. 2h). The results of Pearson analyses showed that the Krt5^+^ area was positively correlated with the pancreatic inflammation score (Fig. 2i), acinar cell necrosis score (Fig. 2j) and pathologic score (Fig. 2k). These results suggested that Krt5^+^ cell activation in ANP is closely associated with the pancreatic injury.

### Persistent Notch activity in ANP pancreases correlates with the formation of duct-like structures

**Fig. 3:**
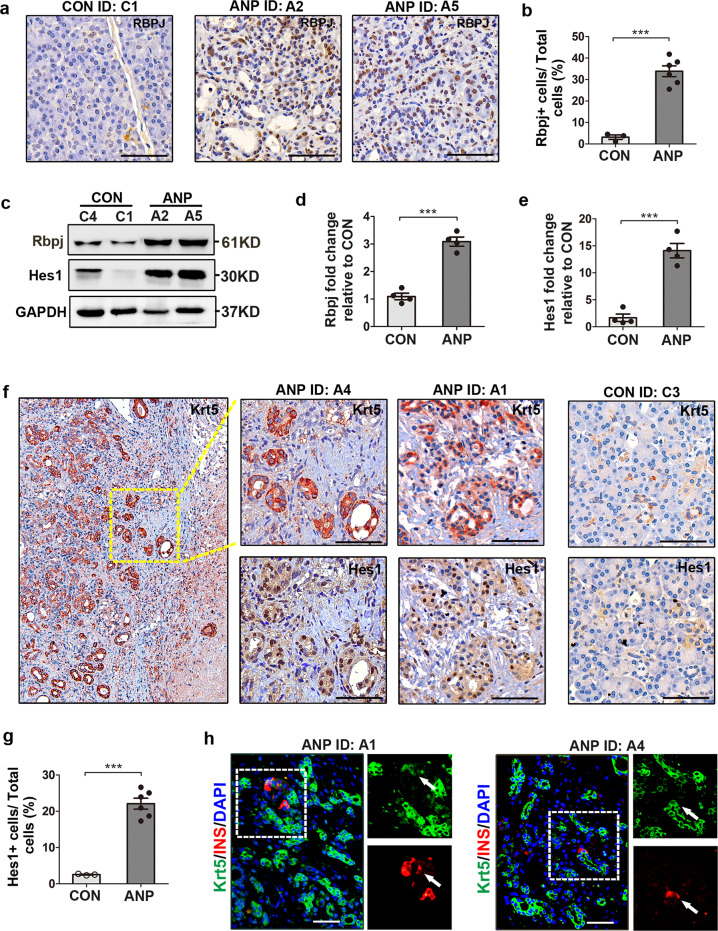
Persistent Notch activity in ANP pancreases correlates with Krt5^+^ colonies and duct-like structures. **a**, **b** Representative staining and quantification of Rbpj expression in pancreases of control and ANP individuals. **c** Representative western blots of Rbpj and Hes1 in pancreases from control individuals and ANP patients. **d**, **e** Quantification of western bolts for Rbpj (**d**) and Hes1 (**e**) in ANP pancreases compared with that in control pancreases. **f** Representative images of serial sections stained with Krt5 and Hes1 showing that Krt5^+^ duct-like or clustered structures were also immunopositive to nuclear Hes1. **g** Quantification of Hes1^+^ cells divided by total cells (shown as %), plotted as fold change vs control. **h** Images of pancreases from ANP individuals immunostained with Krt5 (green), insulin (red) and DAPI (blue) showing seldomly noticed double-labeling cells (white arrowhead). ANP, acute necrotic pancreatitis; CON, control. Data are mean ± SEM, *n* = 3–6. ***p < 0.001. Scale bars, 50 μm.

Notch signaling is responsible for the expansion of Krt5^+^ progenitors in lung injury. Therefore, we assessed the magnitude of Notch activation in ANP pancreases. While low levels of Rbpj were detected in pancreatic sections from controls, strong Rbpj immunoreactivity was detected in ANP pancreases (Fig. 3a, b). In contrast to controls, the protein levels of Rbpj and Hes1 increased significantly in ANP pancreases (Fig. 3c–e). To determine the extent of Notch activity in Krt5^+^ cells, serial sections were used to assess the localization of Hes1 in relation to Krt5. Interestingly, the Krt5^+^ duct-like or clustered structures were also immunopositive for nuclear Hes1, which was notably more intense in pancreatic sections from ANP patients than in controls (Fig. 3f, g). To examine whether Krt5^+^ cells could differentiate into beta cells in ANP patients, immunofluorosent double-labeling of Krt5 and insulin was performed. Krt5^+^ cells with immunoreactivity to insulin were seldom noticed (Fig. 3h). Thus, persistent Notch activity prohibited Krt5^+^ cell differentiation in lung regeneration, which might also explain the limited differentiation of Krt5^+^ cells into beta cells, as well as numerous duct-like and clustered structures in pancreases from ANP patients.

### Beta-cell loss in murine AP model and massive Krt5^+^ cell activation in the pancreas of AP mice

**Fig. 4:**
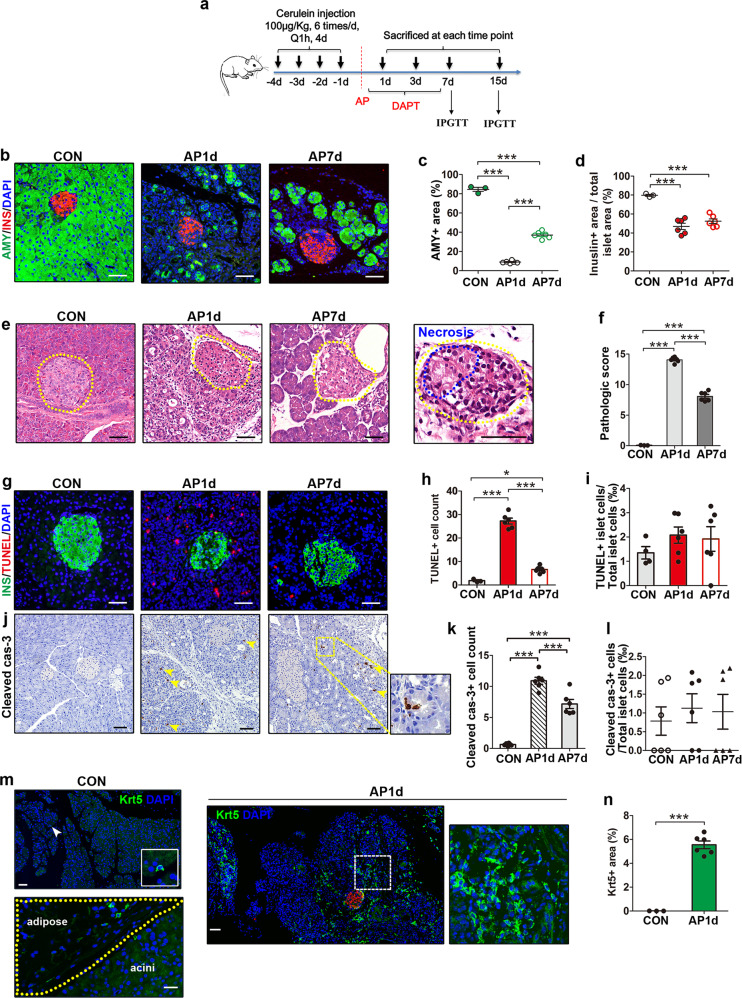
Beta-cell loss in murine AP model and massive Krt5^+^ cells activation in pancreases of AP mice. **a** Schematic depicting the study protocol of animal experiments. **b** Images of pancreases from control and AP mice immunostained with amylase (green), insulin (red) and DAPI (blue). **c** Quantification of pancreatic area immunostained positive for amylase (shown as %). **d** Quantification of islet area labeled with insulin divided by total islet area (shown as %) in pancreatic sections from control and AP mice at indicated time points. **e** Representative magnification of H&E staining of pancreases from control and AP mice at indicated time points. **f** Pathologic scoring of pancreatic injury in pancreases from control and AP mice. **g** Representative images of pancreases from control and AP mice immunostained for TUNEL (red), insulin (green), and DAPI (blue). **h** Quantification of TUNEL^+^ cells in pancreases from control and AP mice (×200 magnification). **i** Quantification of TUNEL^+^ cells in islets divided by total islet cells (shown as ‰) in pancreases from control and AP mice. To perform TUNEL^+^ cell counting in islets, ten islets were randomly determined across each section. **j** Immunostaining of cleaved caspase-3 in control and AP pancreases. Arrowheads point to cleaved caspase-3^+^ cells. **k** Quantification of cleaved caspase-3^+^ cells in control and AP pancreases under ×200 magnification. **l** Quantification of cleaved caspase-3^+^ cells in islets divided by total islet cells (shown as ‰) from control and AP mice. To perform cleaved caspase-3^+^ cell counting in islets, ten islets were randomly determined across each section. **m** Representative images of pancreases from control mice stained for Krt5 (green) and DAPI (blue) showing rare Krt5^+^ cells in the stromal part of pancreases and peripancreatic adipose tissue. Images of pancreases from AP mice immunostained with Krt5 (green) and DAPI (blue) showing the broad distribution of Krt5^+^ cells. **n** Quantification of the area distributed with Krt5^+^ cells in pancreases from control and AP mice, divided by the total area (shown as %). AMY, amylase; AP, acute pancreatitis; Con, control; INS, insulin. Data are mean ± SEM, *n* = 3–6. *p < 0.05, ***p < 0.001. Scale bars, 50 μm.

To examine pancreatic injury and beta-cell loss in the AP model, we performed immunofluorescent staining with amylase and insulin. A significantly decreased amylase-positive acinar area following AP was consistent with acinar destruction, as observed by H&E staining (Fig. 4b, c, e). Immunostaining against insulin revealed that the ratio of beta-cell area decreased significantly one day following AP, which indicated beta-cell loss. Unlike the rapidly regenerated exocrine counterparts 7 days after AP, beta cells did not strongly recover since the ratio of insulin-positive area to total islet area did not increase significantly compared to the AP1d group (Fig. 4d). Similar to ANP human pancreases, compared to normal pancreatic structures and integral islets in the controls, increased acinar necrosis, inflammatory infiltration and obvious cell death features inside islets were observed in the AP1d group (Fig. 4e, f). While the exocrine structure gradually recovered at 7 days postinjury, some islets with cell necrosis features remained in severely injured areas (Fig. 4e). Similar to that of ANP patients, the TUNEL assay demonstrated increased apoptosis in the exocrine pancreas but not in islets following AP (Fig. 4g–i). Immunostaining of cleaved caspase-3 revealed similar results (Fig. 4j–l). Western blotting analysis also showed that the protein levels of cleaved caspase-3 in islets isolated from AP and control mice were barely detectable, and did not differ (Supplementary Fig. 3). In contrast to the rarely seen Krt5^+^ cells in the stromal part and peripancreatic adipose tissue of control pancreases, pancreatic sections of AP1d mice revealed a broad distribution of Krt5^+^ cells in the damaged regions (Fig. 4m, n).

### Inhibition of Notch activity improves glucose tolerance in AP mice

**Fig. 5:**
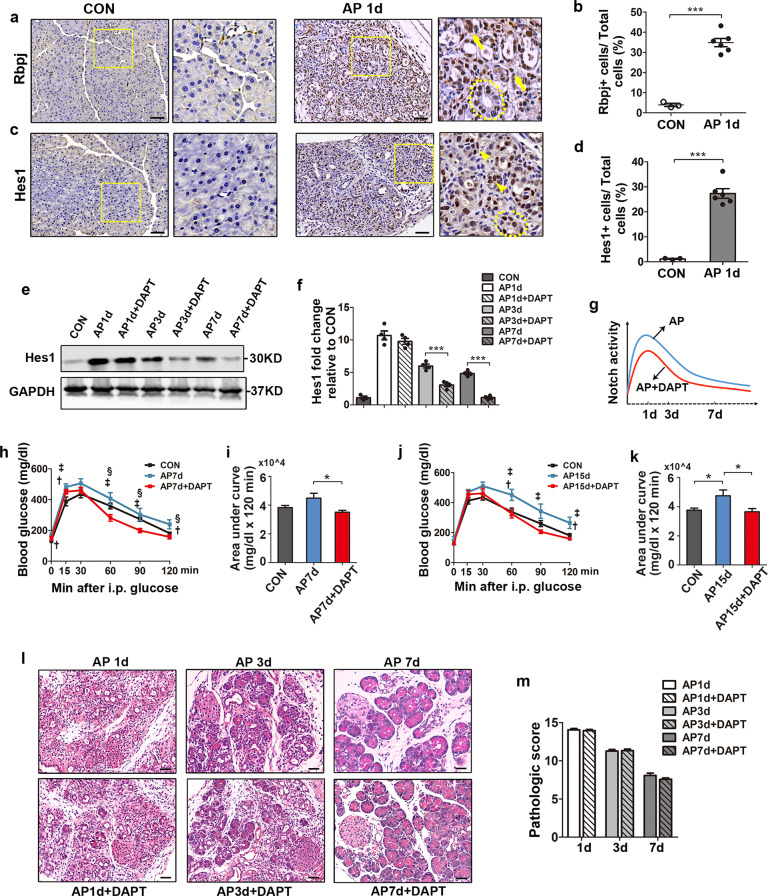
Notch inhibition improves glucose tolerance in AP mice. **a** Staining of Rbpj in pancreases of control and AP1d mice showing prominent Rbpj immunoreactivity in pancreases of AP mice. **b** Quantification of Rbpj^+^ cells (shown as %) in pancreases from control and AP1d mice. **c** Immunostaining of Hes1 in pancreases of control and AP1d mice showing prevalent Hes1 staining in pancreases of AP1d mice compared with control pancreases. **d** Quantification of Hes1^+^ cells (shown as %) in pancreases from control and AP1d mice. **e** Representative western blots of Hes1 in pancreases from control mice, AP mice and AP mice treated with DAPT. **f** Quantification of western blots for Hes1 in pancreases from AP mice and AP mice treated with DAPT in relative to control mice. **g** Schematic representation of Notch activity in pancreases from AP mice and mice treated with DAPT at indicated time points. **h**, **i** Blood glucose levels and AUCs during the IPGTT performed in AP7d mice and corresponding DAPT-treated mice, in comparison with control mice (*n* = 7, 7 and 10 in CON, AP7d and AP7d + DAPT group, respectively; ^†^*p* < 0.05, CON vs AP7d; ^‡^*p* < 0.05, CON vs AP7d + DAPT; ^§^*p* < 0.05, AP7d vs AP7d + DAPT). **j**, **k** Blood glucose levels and AUCs during the IPGTT performed in AP15d mice and corresponding DAPT-treated mice compared with that of control mice (*n* = 6, 7 and 8 in CON, AP15d and AP15d + DAPT group, respectively; ^†^*p* < 0.05, CON vs AP15d; ^‡^*p* < 0.05, AP15d vs AP15d + DAPT). **l**, **m** Representative H&E staining and pathologic scoring of pancreases from control mice, AP mice and DAPT-treated mice at indicated time points. AP, acute pancreatitis; CON, control; i.p., intraperitoneal; IPGTT, intraperitoneal glucose tolerance test. Data are mean ± SEM, *n* = 3–6. *p < 0.05, ***p < 0.001. Scale bars, 50 μm.

As a first step to evaluating Notch activity in mouse pancreases, we examined the expression of Rbpj and Hes1 in both the control and AP groups. Prevalent Rbpj and Hes1 staining in pancreatic sections from AP mice were observed compared with controls (Fig. 5a–d). Treatment with DAPT reduced Notch activity at the indicated time points compared with the corresponding AP groups, as shown by the protein levels of Hes1 (Fig. 5e–g). We next evaluated whether Notch activity contributed to endocrine dysfunction of the pancreas. Compared with the controls, mice in the AP7d and AP15d groups showed impaired glucose tolerance and increased area under the curve (AUC) in IPGTT tests (Fig. 5h–k). However, impaired glucose tolerance and elevated AUC were reversed with DAPT administration, suggesting that Notch signaling inhibition could improve glucose tolerance in the AP model. To evaluate the effects of Notch activity on the exocrine pancreas, we analyzed H&E staining of each group and found no difference in pancreatic injury between the AP and DAPT-treated groups (Fig. 5l, m).

### Notch signaling inhibition in AP enhances the differentiation of Krt5^+^ cells into functional beta cells

**Fig. 6:**
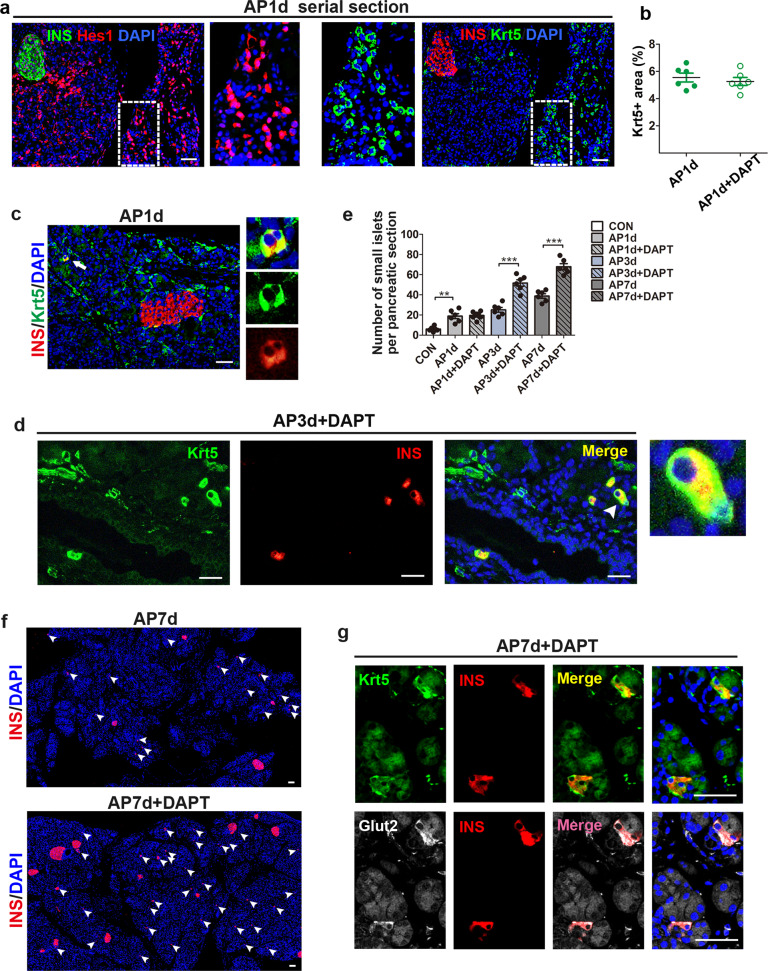
Notch inhibition following AP promotes differentiation of Krt5^+^ cells to functional beta cells. **a** Immunostaining of serial sections probed with insulin (green, left), Hes1 (red), insulin (red, right) and Krt5 (green) showing Krt5^+^ cells were also immunopositive to nuclear Hes1, nuclei were stained with DAPI (blue). **b** Quantification of area covered with Krt5^+^ cells in pancreases of AP1d and corresponding DAPT-treated mice. **c** Representative images of pancreases from AP1d mice stained with Krt5 (green), insulin (red) and DAPI (blue) showing double-positive Krt5^+^ and insulin^+^ cells, indicating that Krt5^+^ cells could differentiate to beta cells. **d** Representative graphs of pancreases form AP3d + DAPT mice stained with Krt5 (green), insulin (red) and DAPI (blue). **e** Quantification of newborn small islets in pancreases from control mice, AP mice and DAPT-treated mice at indicated time points. **f** Immunofluorescent images of pancreases stained with insulin (red) and DAPI (blue) showing increased numbers of newborn small islets in AP7d + DAPT pancreases compared with that of AP7d group. White arrowheads point to the newborn small islets. **g** Representative images of serial sections immunostained with Krt5 (green), insulin (red), Glut-2 (white) and DAPI (blue) showing small islets stained positive with Krt5 were also immunopositive for Glut2, suggesting the small islets differentiated from Krt5^+^ cells were functional. AP, acute pancreatitis; CON, control; INS, insulin. Data are mean ± SEM, *n* = 6. **p *<* 0.01, ***p < 0.001. Scale bars, 50 μm.

To determine the activation of Notch signaling in Krt5^+^ cells, serial sections were used to assess the localization of Hes1 in relation to Krt5 in AP1d pancreases by immunofluorescence staining. Similar to the findings in ANP patients, Krt5^+^ cells in AP pancreases showed Hes1 immunoreactivity in the nucleus, suggesting increased Notch activity in Krt5^+^ cells (Fig. 6a). DAPT treatment did not significantly influence the expansion of Krt5^+^ cells one day after AP (Fig. 6b). We next evaluated whether Krt5^+^ cells could differentiate into beta cells by double-labeling with Krt5 and insulin. A tiny proportion of double-positive Krt5^+^ and insulin^+^ cells were already detectable in the AP1d pancreas (Fig. 6c), indicating the differentiation of Krt5^+^ cells into beta cells. Intriguingly, immunostaining of pancreases from the AP3d + DAPT group showed a certain amount of double-labeled Krt5^+^ and insulin^+^ cells (Fig. 6d). To determine the effect of Notch inhibition on the differentiation of Krt5^+^ cells into beta cells, we evaluated the number of small islets in the AP and corresponding DAPT groups. Similar proportions of small islets were exhibited in the AP1d and AP1d + DAPT groups, whereas the numbers of small islets increased significantly in the DAPT-treated groups compared with the corresponding AP groups with the continued administration of DAPT (Fig. 6e), suggesting that more Krt5^+^ cells differentiated into beta cells after Notch inhibition. As shown in Fig. 6f and Supplementary Fig. 4, significantly increased numbers of small islets were noted in the DAPT-treated groups. These results, at least in part, explained the improved glucose tolerance of DAPT-treated groups on days 7 and 15 in the AP model. To test whether the newborn small islets were functional, serial sections were used to locate Krt5, insulin and Glut-2 expression. The small islets differentiated from Krt5^+^ cells were also immunopositive for Glut-2, suggesting that the newborn islets were functional (Fig. 6g).

### Krt5^+^ cells differentiate toward beta cells by Notch signaling inhibition in vitro

**Fig. 7:**
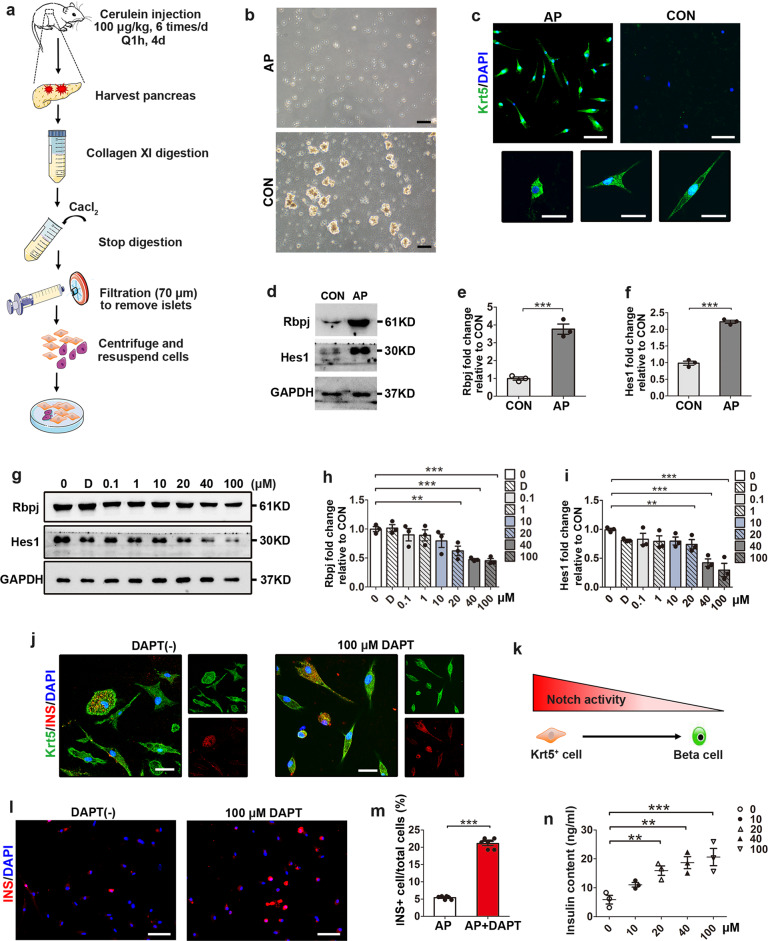
Increased Krt5^+^ cells differentiation toward beta cells following Notch inhibition in vitro. **a** Schematic depicting the protocol of primary cell isolation. **b** Images of primary cells isolated from pancreases of control and AP mice. **c** Identification of Krt5^+^ cells with immunostaining of Krt5 (green) and DAPI (blue) in isolated primary cells from control and AP mice. **d** Representative western blots of Rbpj and Hes1 in isolated primary cells from control and AP mice. **e**, **f** Quantification of western blots for Rbpj (**e**) and Hes1 (**f**) in cells isolated from AP mice vs control mice. **g** Western blot analyses of Rbpj and Hes1 expression in primary cells from AP mice treated with various concentrations of DAPT for 72 h. **h**, **i** Quantification of western blots for Rbpj (**h**) and Hes1 (**i**) in cells isolated from AP mice supplemented with various concentrations of DAPT for 72 h. **j** Representative images of cells with and without DAPT treatment immunostained with Krt5 (green), insulin (red) and DAPI (blue) showing the increased proportion of double-positive Krt5^+^ and insulin^+^ cells, suggesting more Krt5^+^ cells differentiated into beta cells following Notch inhibition. **k** Schematic representation showing Krt5^+^ cells differentiated toward beta cells following downregulation of Notch activity. **l** Images of cells isolated from AP mice supplemented with DAPT showing the increased proportion of insulin^+^ cells after 72 h culture compared with cells without DAPT treatment. **m** Quantification of insulin^+^ cells (shown as %) after 72 h culture with and without DAPT treatment. **n** Measurement of insulin content in cells isolated from AP mice treated with various concentrations of DAPT for 72 h. AP, acute pancreatitis; CON, control; D, DMSO; INS, insulin. Data are mean ± SEM, *n* = 3 experiments; each experiment was performed using cells from independent primary cell isolation. **p < 0.01, ***p < 0.001. Scale bars, 50 μm (**b**, **c** upper graphs, **l**) and 20 μm (**c** lower graphs, **j**).

To isolate the Krt5^+^ cells from the pancreas, mice were killed following AP establishment, and primary cells were isolated (Fig. 7a). In contrast to the cells extracted from control mice, which were mostly clustered acinar cells, cells isolated from AP mice were single cells with few acinar cells (Fig. 7b). This was consistent with the in vivo findings that acinar structures were abrogated in AP mice. To identify the Krt5^+^ cells, immunofluorescent staining of Krt5 was performed and we observed polymorphous Krt5^+^ cells growing on glass slides (Fig. 7c). We extracted cell proteins immediately after isolation and found that cells isolated from the pancreases of AP mice showed significantly higher protein levels of Rbpj and Hes1 than controls (Fig. 7d–f). To perform Notch activity inhibition experiments in vitro, a range of concentrations of DAPT were supplemented. Immunoblotting results showed that cells incubated for 72 h with 20 μM, 40 μM and 100 μM DAPT presented a dose dependent inhibitory effect on Notch activity (Fig. 7g–i; 200 μM DAPT was abandoned due to insolubility). To confirm the differentiation of Krt5^+^ cells into beta cells in vitro, double staining of Krt5 and insulin was performed. Compared with Krt5^+^ cells cultured in regular medium, cells treated with DAPT (100 μM) exhibited an increased proportion of double-positive Krt5^+^ and insulin^+^ cells after 72 h of culture (Fig. 7j). We also calculated the proportion of insulin^+^ cells. Compared with cells cultured in regular medium for 72 h, cells cultured with DAPT (100 μM) for the same period showed a significantly increased proportion of insulin^+^ cells (Fig. 7l, m). Cells treated with DAPT (100 μM) for 72 h also showed increased insulin content, as verified by ELISA (Fig. 7n). The above findings suggested that more Krt5^+^ cells differentiate into beta cells under Notch inhibition in vitro.

## Discussion

**Fig. 8:**
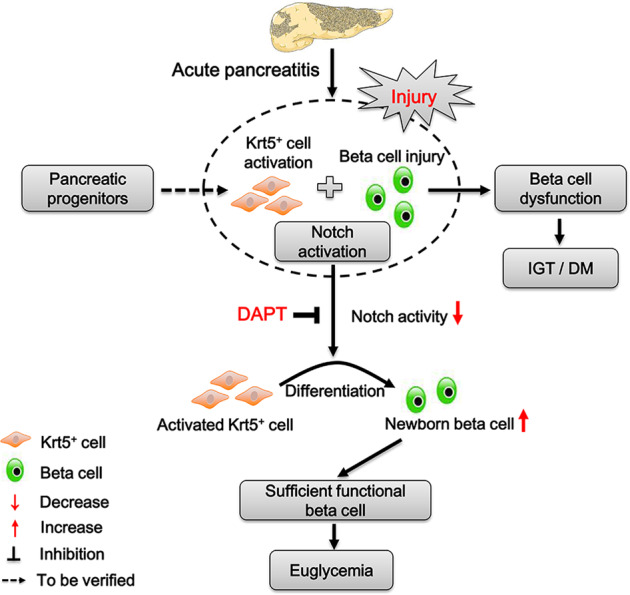
Diagram of potential mechanism of Notch inhibition in pancreatic Krt5^+^ cell differentiation and glucose tolerance restoration following AP. After AP, beta-cell loss induces IGT or even DM. At the same time, Krt5^+^ cells are activated together with upregulated Notch activity. Subsequent Notch inhibition would contribute to the differentiation of Krt5^+^ cells toward functional beta cells, thus restoring the glucose homeostasis. DM, diabetes mellitus; IGT, impaired glucose tolerance.

In the present study, we provided direct evidence of beta-cell loss with ANP human specimens and an AP model, which contributes to pancreatic endocrine insufficiency following AP. Importantly, we established for the first time that Krt5^+^ cells are activated in injured pancreases of ANP patients and AP mice, and can differentiate into functional beta cells. Pancreatic injury could induce massive Krt5^+^ cell activation, which was accompanied by upregulation of Notch activity, whereas subsequent Notch blockade promoted the differentiation of Krt5^+^ cells into functional beta cells (Fig. 8). These results could explain the increased numbers of small islets and improved glucose tolerance after Notch inhibition in the murine AP model. Persistent high levels of Notch signaling in the pancreases of ANP patients led to the formation of massive duct-like structures, which compromised the differentiation of beta cells from Krt5^+^ cells.

Previously, chronic pancreatitis and pancreatic ductal adenocarcinoma were the most commonly identified causes of type 3c diabetes [26, 27]. In recent years, type 3c diabetes following AP has gradually attracted more attention. A recent study indicated that the contribution of AP to the risk of type 3c diabetes was considerably larger (83% versus 17%) than that of chronic pancreatitis [28]. Whether the increased risk of DM in AP patients attributed to beta-cell injury remains unknown. We observed a significantly reduced insulin^+^ area, which represented beta-cell loss inside islets in both human and mouse pancreases. In clinical settings, ANP patients demonstrated varying degrees of necrosis of the pancreas parenchyma, which was accompanied by different levels of beta-cell loss. Although the ANP specimens obtained in our study were fresh tissues adjacent to the surgical margin, we still noticed the manifestations of necrosis in islets upon H&E staining. A reasonable explanation might be that, in contrast to the chronic damage and mild injury to beta cells in type 1 or type 2 diabetes, AP usually exhibits a more aggressive process, which leads to extreme biochemical stress and uncontrolled beta-cell death. In line with this, AP patients develop prediabetes or even diabetes when beta-cell function cannot compensate for such irreversible beta-cell loss. In addition, we also observed increased autophagic vacuoles in beta cells of ANP patients under electron microscopy. Increased autophagy has been suggested to be associated with loss of beta-cell mass in diabetic patients [29]. In another study, autophagy activation is proposed to be related to increased insulin sensitivity and islet beta-cell proportion in a high-fat diet model [30]. Thus, the roles of altered autophagy in beta cells after AP remain to be examined. Available data demonstrated apoptosis as a main type of beta-cell death in type 1 and type 2 diabetes [31–34]. In our study, we observed significantly increased apoptosis in the exocrine pancreas but not in the islets following AP. Thus, whether there are differences in the cell death forms of beta cells between type 1/2 diabetes and AP needs to be explored in the future. Although we revealed an increased percentage of alpha cell area divided by total islet area in the remaining pancreas of ANP patients, considering the islet loss due to pancreas necrosis, the number of total alpha cells remains to be verified.

The progenitor-like properties of Krt5^+^ cells have been demonstrated in salivary organogenesis and lung regeneration [10, 35, 36]. The results of this study verified the activation of Krt5^+^ cells in pancreases of ANP patients and AP mice. A positive correlation between Krt5^+^ cell activation and pancreatic injury has also been revealed, which was consistent with the previous notion that the activation of progenitor cells or mesenchymal stem cells might depend on the levels of inflammation within the residing tissues [37]. However, unlike the interstitial distribution pattern of Krt5^+^ cells in the injured mouse pancreas, we observed large quantities of Krt5^+^ duct-like structures or clusters that also stained positive for Hes1 in ANP pancreases. This was because the pancreatic specimens from ANP patients suffered uncontrollable peripancreatic infection for several weeks. The persistent infection, inflammation and injury of pancreases in ANP patients resulted in hyperactivity of Notch signaling, contributing to the maintenance of Krt5^+^ cell identity and impeding their differentiation into beta cells, ultimately facilitating the formation of Krt5^+^ ducts and clusters. As reported by Vaughan in their study, persistent Notch signaling prevented Krt5^+^ cells from differentiating into alveolar cell types and led to the formation of honeycomb cysts in the lung, while removal of Notch signaling promoted Krt5^+^ cell differentiation into alveolar type II cells [35]. A study in pancreas development confirmed that overexpression of Notch signaling prevented differentiation and trapped cells in the progenitor state [19, 38]. Research in zebrafish revealed that endocrine differentiation, including beta-cell linage differentiation, required strong Notch signaling downregulation [39]. The Krt5^+^ duct-like structures formed in pancreatitis might resemble acinar-to-ductal metaplasia (ADM), which contributes to the formation of premalignant lesions if persistent for a long time [40]. This might explain why postpancreatitis diabetes mellitus poses a high risk for pancreatic cancer [41]. This perspective needs to be verified in the future study. In this study, double-positive Krt5^+^ and insulin^+^ cells indicated the differentiation process of Krt5^+^ cells into beta cells. The Notch inhibitor application significantly increased the number of small islets and reversed the impaired glucose tolerance following AP in our murine model, which was to some extent attributed to the enhanced differentiation of Krt5^+^ cells toward beta cells. In addition, in vitro experiments further confirmed the observation of Krt5^+^ cells differentiating into beta cells as well as increased beta cells following DAPT treatment. Since the lineage tracing method was not employed in this study, whether the Krt5^+^ cells are differentiated from other cell lineages or are inherently present in the pancreas remains to be determined. Besides Notch signaling, other important mechanisms in stem cell differentiation including Wnt and Hedgehog pathways, as well as autophagy, might also affect Krt5^+^ differentiation. For example, autophagy has been indicated to be required for maintaining the stemness and differentiation of stem cells [42, 43].

In summary, our findings suggest that beta-cell loss following AP contributes to pancreatic endocrine dysfunction. We have demonstrated the existence of Krt5^+^ cells, which are activated in injured pancreases of both human ANP and murine AP model. We highlight that appropriate Notch inhibition could promote the differentiation of Krt5^+^ cells toward functional beta cells, which to some extent explains the increased number of small islets and improved glucose tolerance in AP mice. The findings from this study could provide a new strategy for the treatment of prediabetes/diabetes following AP.

## Supplementary information

Supplementay materials

Supplementary Fig. 1

Supplementary Fig. 2

Supplementary Fig. 3

Supplementary Fig. 4

## Data Availability

The data presented in this manuscript are available upon reasonable request from the corresponding authors.
