# Supplementary material for: Inhibition of Notch activity promotes pancreatic cytokeratin 5-positive cell differentiation to beta cells and improves glucose homeostasis following acute pancreatitis
Source: Cell Death Dis. 2021 Sep 23;12(10):867. doi: 10.1038/s41419-021-04160-2 (PMC8460737; doi:10.1038/s41419-021-04160-2)
Supplement: Supplementary file 1 — Supplementay materials [file 41419_2021_4160_MOESM1_ESM.docx]

**Supplementary materials**

**Supplementary Table 1**

**Clinical characteristics of ANP patients and non-ANP individuals (CON)**

| Patient ID | Age | Sex | BMI (kg/m^2^) | Diagnosis | Time from ANP onset to operation | Outcome |
| --- | --- | --- | --- | --- | --- | --- |
| ANP group |  |  |  |  |  |  |
| A1 | 49 | M | 31.1 | ANP | 34d | Survival |
| A2 | 40 | M | 26.4 | ANP | 21d | Death |
| A3 | 46 | M | 26.7 | ANP | 44d | Death |
| A4 | 65 | M | 26 | ANP | 30d | Death |
| A5 | 65 | M | 22.8 | ANP | 52d | Survival |
| A6 | 66 | F | 28.1 | ANP | 24d | Death |
| Control group |  |  |  |  |  |  |
| C1 | 21 | M | 21.3 | Pancreatic rupture | NA | Survival |
| C2 | 43 | M | 26 | Pancreatic rupture | NA | Survival |
| C3 | 51 | M | 18.7 | DPA | NA | Survival |
| C4 | 26 | F | 20.2 | SPN | NA | Survival |
| C5 | 33 | M | 22.8 | DPA | NA | Survival |
| C6 | 41 | M | Not known | Donar | NA | Death |

ANP, acute necrotizing pancreatitis; BMI, body mass index; CON, control; DPA, duodenal papillary adenocarcinoma; F, female; M, male; NA, not applicable; SPN, solid pseudopapillary neoplasm.

**Supplementary Fig. 1**

**
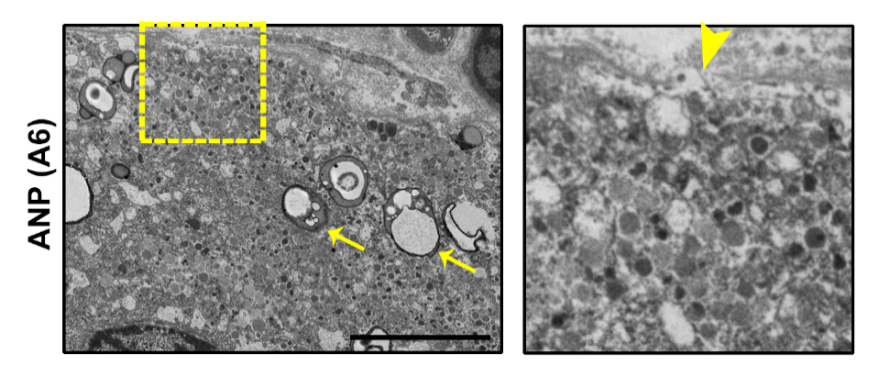
**

**Supplementary Fig. 1** Representative electron microscopy graphs showing dismantled cell membrane and vacuolization of beta cell in the pancreas of ANP patient. Arrows point to autophagic vacuoles; arrowhead points to dismantled cell membrane. ANP, acute necrotizing pancreatitis. Scale bar, 5 μm.

**Supplementary Fig. 2**

**
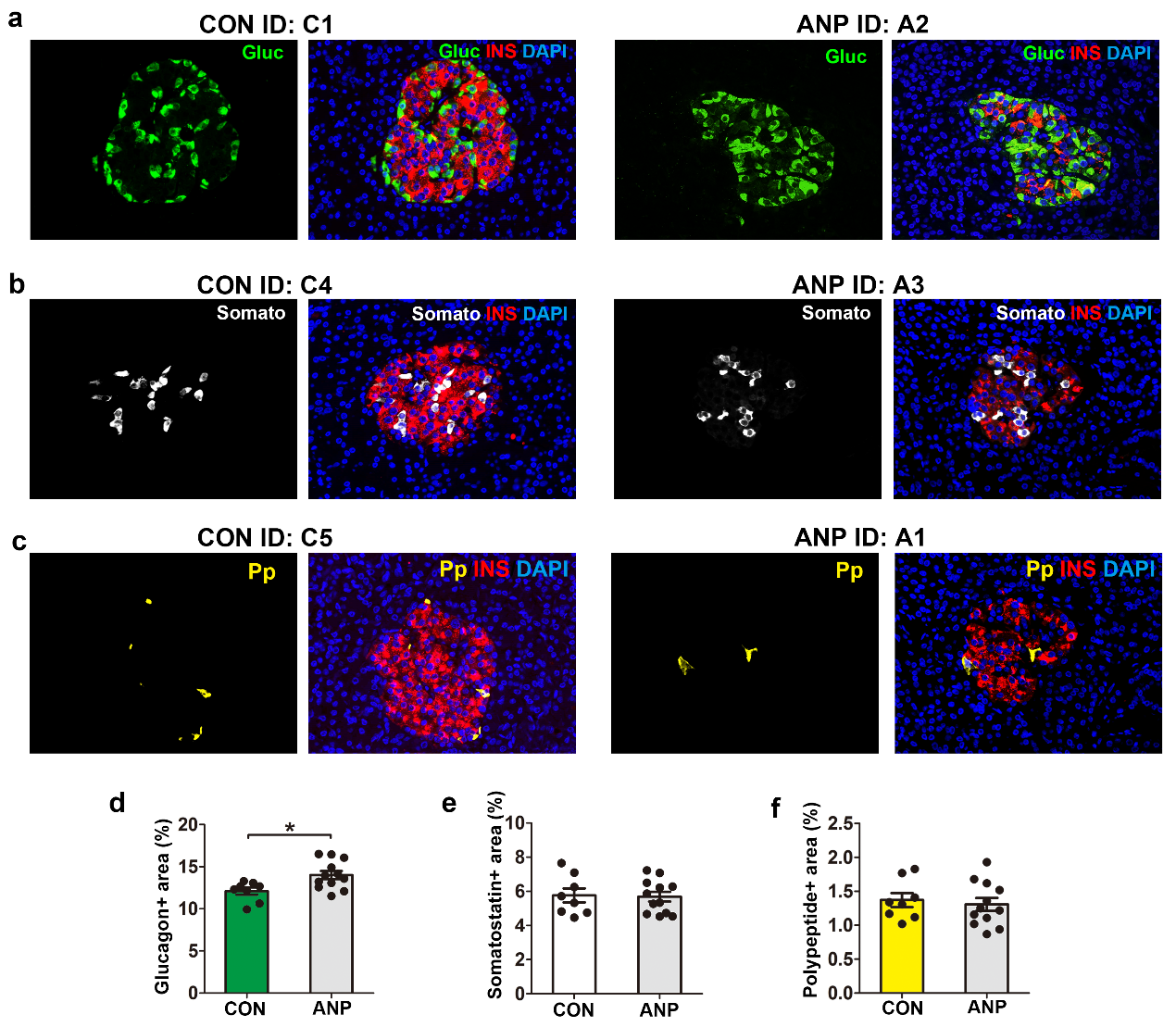
**

**Supplementary Fig. 2 Staining of alpha cells, delta cells and pancreatic polypeptide cells in control and ANP pancreases.** **(a)** Immunofluorescent graphs of pancreases from control and ANP individuals immunostained for glucagon (green), insulin (red) and DAPI (blue). **(b)** Representative staining of pancreases from control and ANP individuals with somatostatin (white), insulin (red) and DAPI (blue). **(c)** Representative images of pancreases from control individuals and ANP patients, stained for pancreatic polypeptide (yellow), insulin (red) and DAPI (blue). **(d), (e)** and **(f)**, quantification of islet area labeled with glucagon, somatostatin, and pancreatic polypeptide divided by total islet area (shown as %) in pancreatic sections from control and ANP individuals. To perform labeling area quantification, pancreatic tissues were scanned across the sections from left to right (2 sections per individual), and an average of 18.15±1.07, 19.05±1.66, 18.75±1.54 islets were analyzed per section for the calculation of glucagon^+^ area, somatostatin^+^ area and Pp^+^ area, respectively. ANP, acute necrotizing pancreatitis; CON, control; Gluc, glucagon; INS, insulin; Pp, pancreatic polypeptide; Somato, somatostatin. Data are mean ± SEM, n=4-6. ^***^*p*＜0.001. Scale bars, 50 μm.

**Supplementary Fig. 3**

**
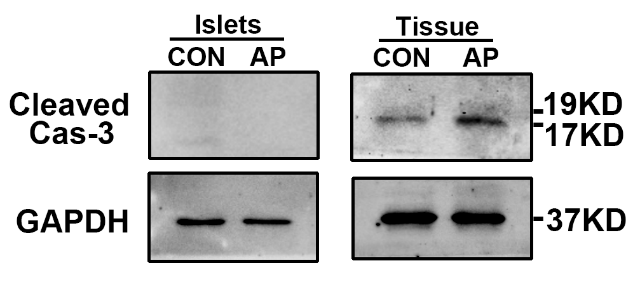
**

**Supplementary Fig. 3 Islets isolated from AP and control mice barely expressed detectable cleaved caspase-3.** Representative western blots of cleaved caspase-3 in islets isolated from AP and control mice, and cleaved caspase-3 in pancreatic tissues of AP and control mice. AP, acute pancreatitis; CON, control.

**Supplementary Fig. 4**

**
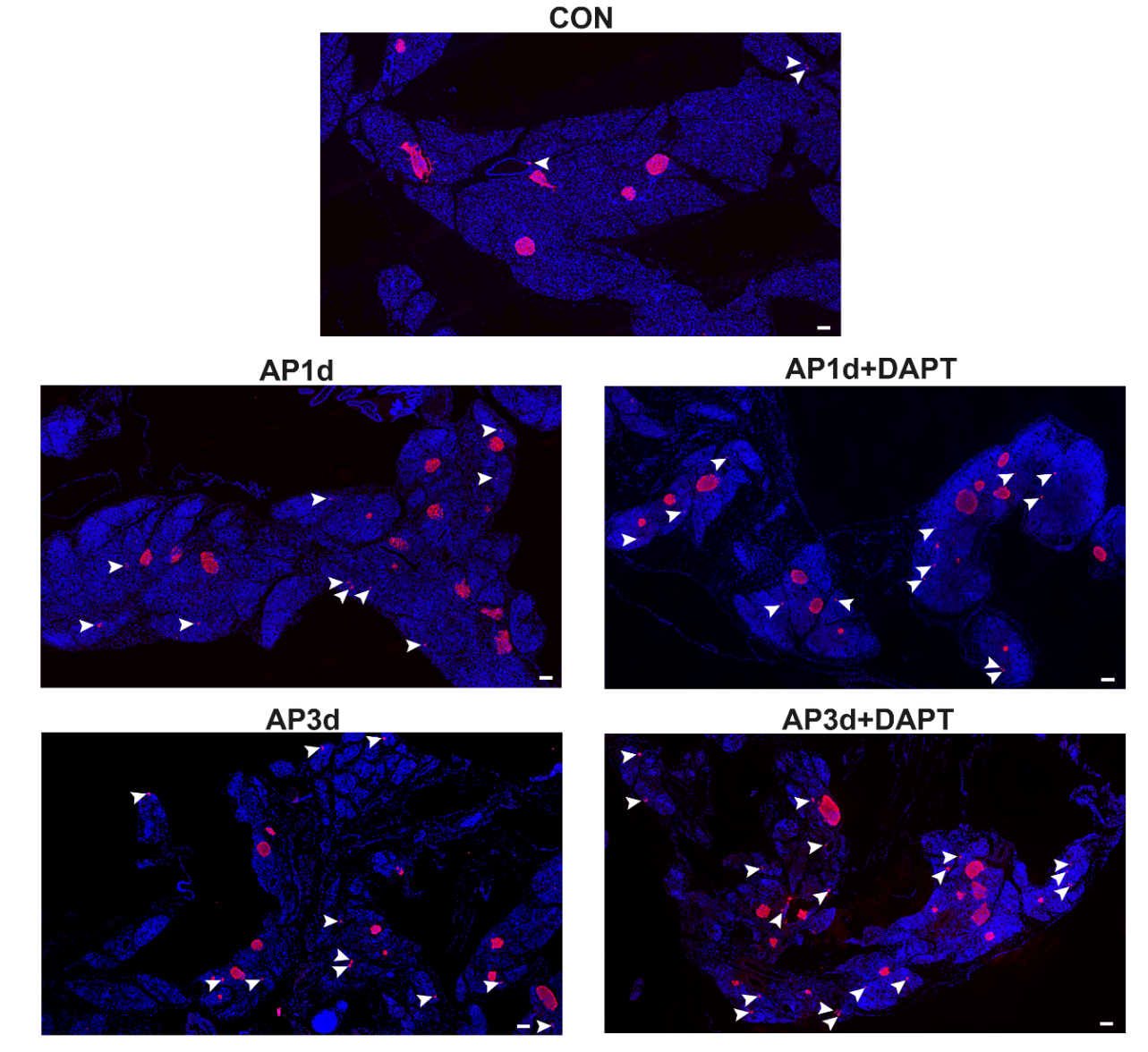
**

**Supplementary Fig. 4** Immunofluorescent images of pancreases stained with insulin (red) and DAPI (blue) showing newborn small islets. AP, acute pancreatitis; CON, control. Scale bars, 100 μm.
