# Supplementary figures and images for: Inhibition of Notch activity promotes pancreatic cytokeratin 5-positive cell differentiation to beta cells and improves glucose homeostasis following acute pancreatitis
Source: Cell Death Dis. 2021 Sep 23;12(10):867. doi: 10.1038/s41419-021-04160-2 (PMC8460737; doi:10.1038/s41419-021-04160-2)

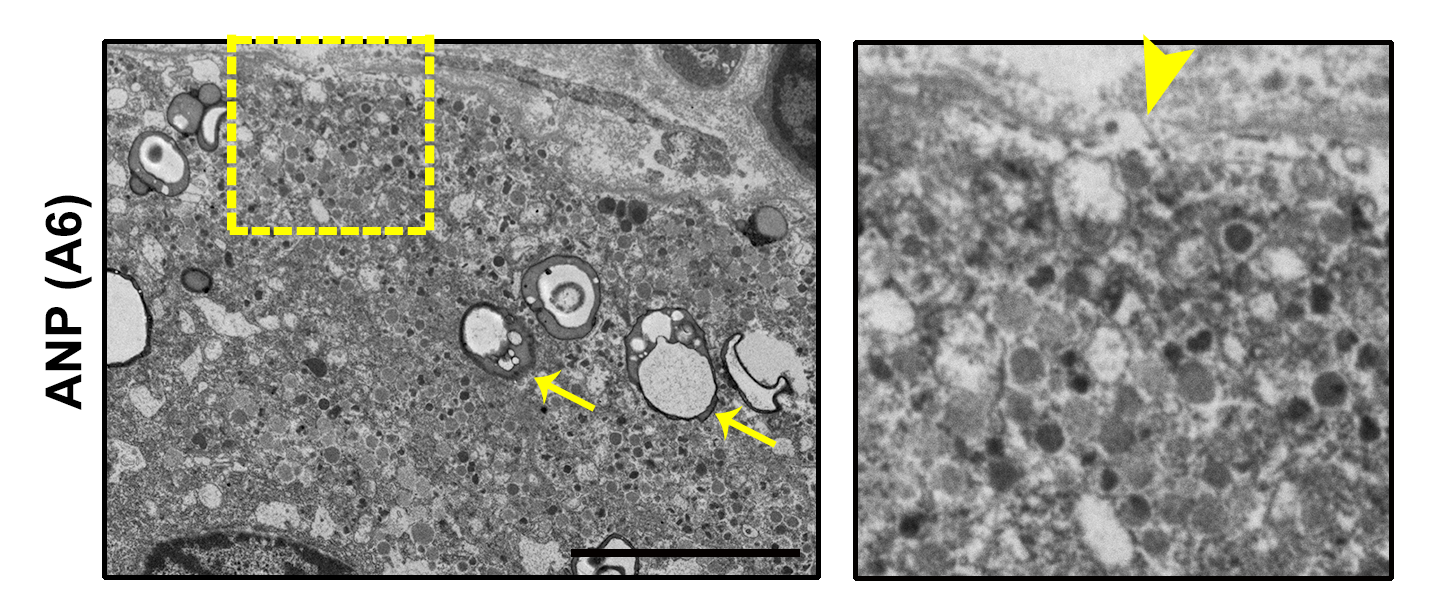

Supplement: Supplementary file 2 — Supplementary Fig. 1 [file 41419_2021_4160_MOESM2_ESM.tif]

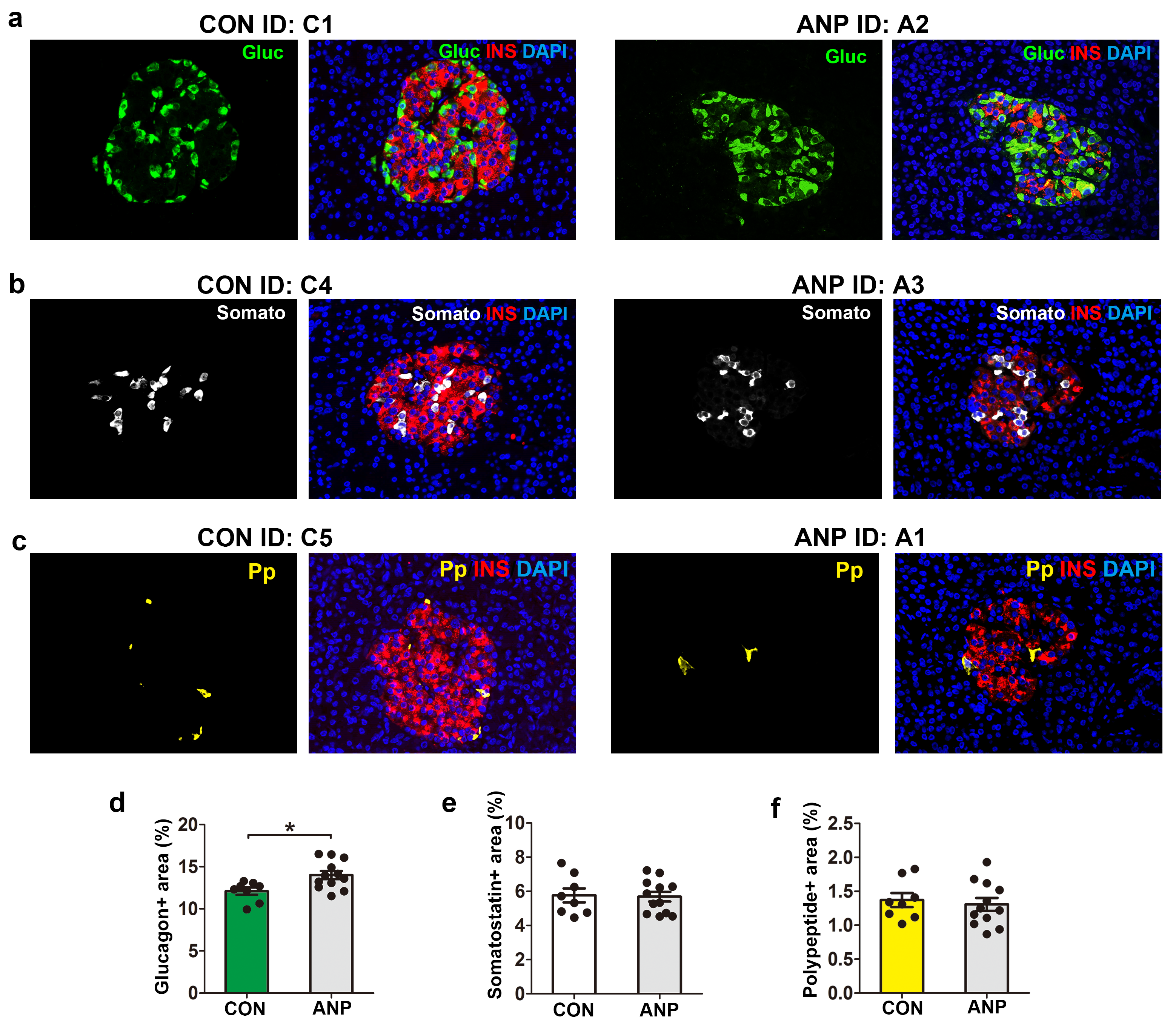

Supplement: Supplementary file 3 — Supplementary Fig. 2 [file 41419_2021_4160_MOESM3_ESM.tif]

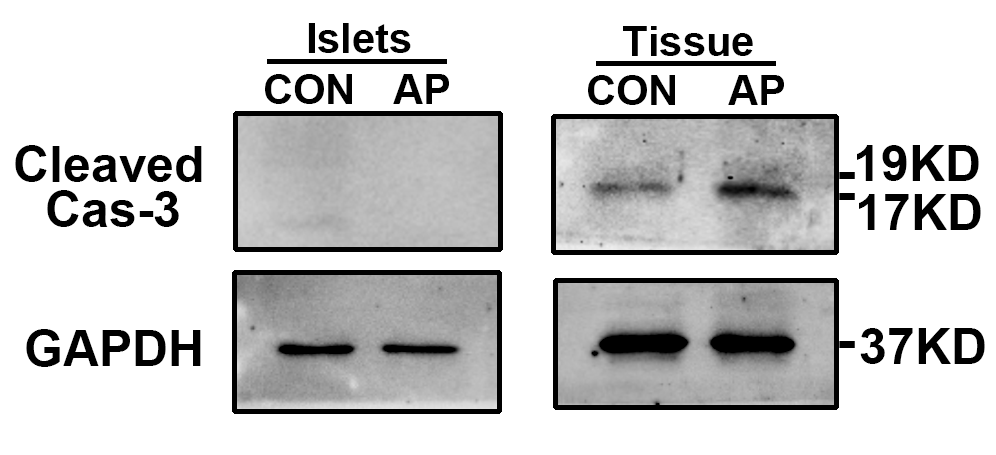

Supplement: Supplementary file 4 — Supplementary Fig. 3 [file 41419_2021_4160_MOESM4_ESM.tif]

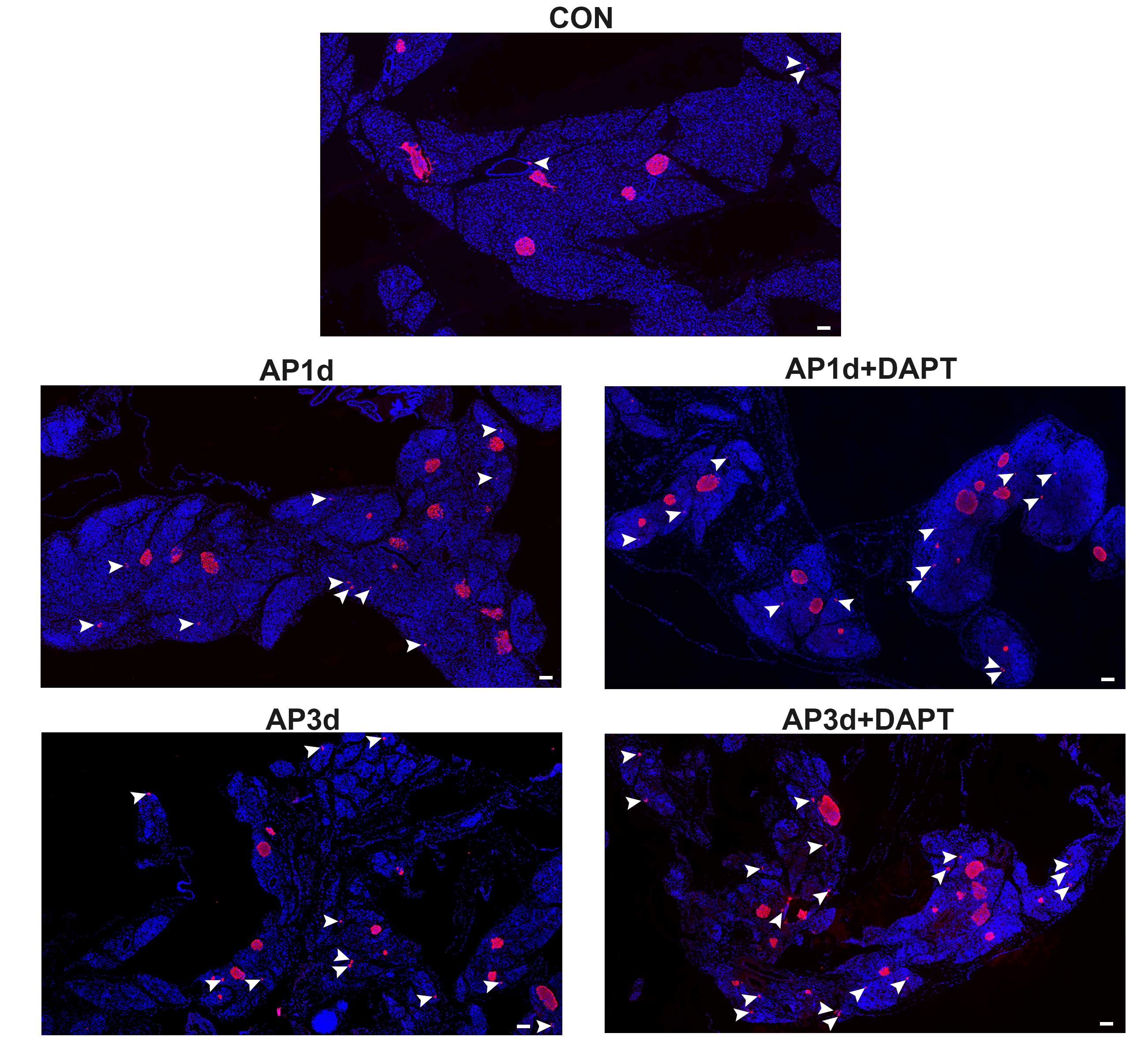

Supplement: Supplementary file 5 — Supplementary Fig. 4 [file 41419_2021_4160_MOESM5_ESM.tif]
